# Fenestration of the Proximal Left Ovarian Vein

**DOI:** 10.7759/cureus.2343

**Published:** 2018-03-19

**Authors:** Mayank Patel, Joe Iwanaga, Rod J Oskouian, R. Shane Tubbs

**Affiliations:** 1 Clinical Anatomy Research, Seattle Science Foundation; 2 Seattle Science Foundation; 3 Neurosurgery, Swedish Neuroscience Institute; 4 Neurosurgery, Seattle Science Foundation

**Keywords:** anatomy, dissection, ovarian vein, gonadal vein, obstetrics and gynecology

## Abstract

A fenestration in the left ovarian vein was found in a fresh-frozen female cadaver. The opening did not have any vessels or additional anatomical structures passing through it. The ovarian vein is also referred to as the female gonadal vein. This type of anatomical variation is clinically relevant in procedures that deal with the manipulation of the gonadal veins, specifically conditions such as ovarian vein thrombosis, ovarian vein stenosis, and pelvic congestion syndrome.

## Introduction

Ovarian veins arise from a venous plexus that covers each ovary. This plexus is known as the pampiniform plexus. The ovarian vein then travels with the ovarian artery, initially through the mesovarium of the broad ligament and then continues through the suspensory ligament. The vein courses anterior to the psoas major muscle after exiting the broad ligament. It is common for two veins to arise from the pampiniform plexus and then reunite into a single vessel to drain into the renal vein on the left side and the inferior vena cava on the right side [1]. Reviewing reports from the literature in regards to this variation, Tubbs et al. mentioned that the gonadal veins may partially divide into two portions to provide a path for the gonadal arteries. The authors also mention that the ovarian vein could present as “several vessels or may form a plexus" [2]. However, we now report an unusual proximal fenestration through the ovarian vein which, to our knowledge, has not been previously reported.

## Case presentation

**Figure 1:**
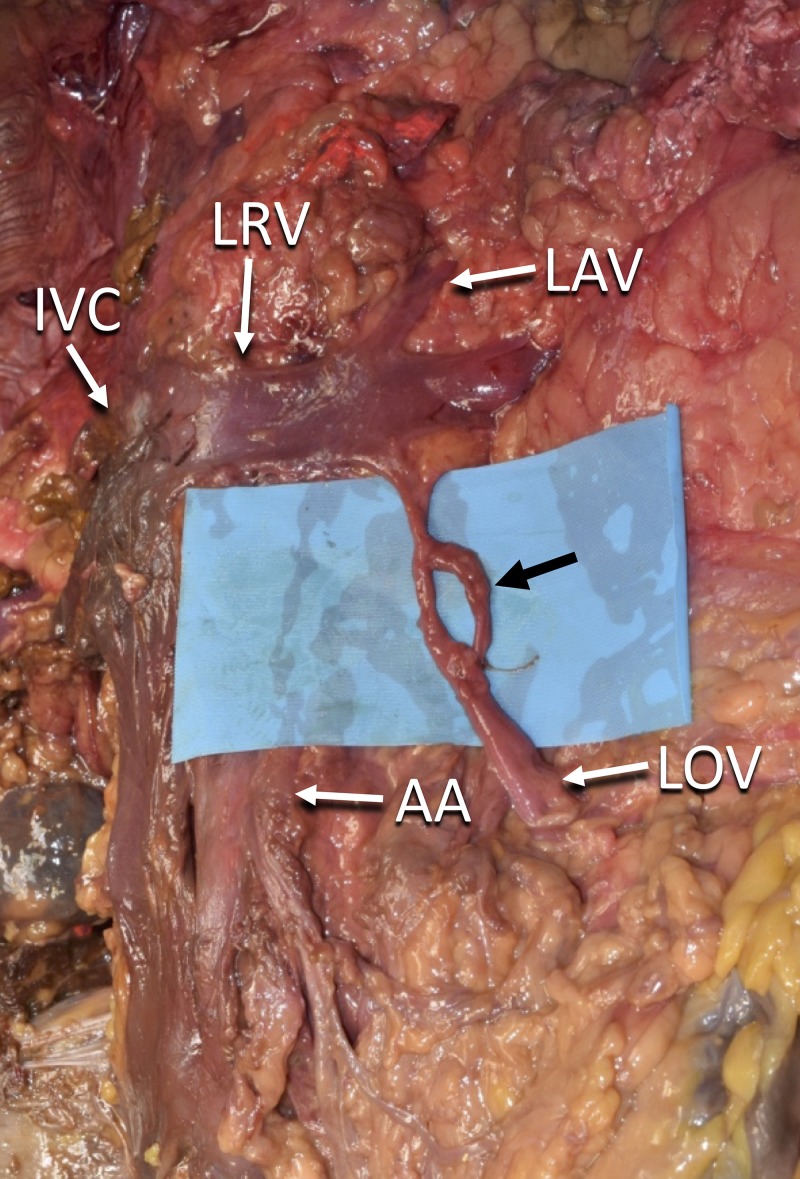
Fenestration of the proximal left ovarian vein Fenestrated left ovarian vein (black arrow); Left ovarian vein (LOV) before the fenestration; Inferior vena cava (IVC); Left renal vein (LRV); Left suprarenal vein (LAV); Abdominal aorta (AA)

During the routine dissection of the abdomen and pelvis in a fresh-frozen, 72-year-old at death, Caucasian, female cadaver, fenestration of the proximal left ovarian vein was identified (Figure 1). The left ovarian vein drained into the left renal vein with course anterior to the descending abdominal aorta and drained into the inferior vena cava (Figure 1). Approximate measurements were made of the dissection, which included the diameter of the fenestration at 0.5 cm, the distance of the fenestration to its drainage into the left renal vein at 1.3 cm, and the distance from the origin of the ovarian vein to the fenestration at 2.12 cm. There were no additional anatomical structures that passed through this left ovarian vein fenestration. No other anatomical anomalies were noted in the specimen relating to the right ovarian vein or abdominopelvic structures. No gross findings of previous surgical intervention to the dissected region were identified.

## Discussion

Recognizing this variation might be important for surgical procedures of the female pelvis. There are conditions such as ovarian vein thrombosis (OVT), ovarian vein stenosis, and pelvic congestion syndrome where understanding the anatomy of the ovarian vein has clinical application. The fenestrated ovarian vein, as seen in our case report, should be considered due to the implications and changes in treatment it may present within the pelvic conditions mentioned here.

OVT is clotting of blood that occurs in the ovarian vein or in nearby draining veins. Computed tomography (CT) and magnetic resonance imaging (MRI) are the preferred imaging modalities for confirming OVT [3]. In a patient with a fenestrated ovarian vein suspected of OVT, the clinician should rule out thromboses in both branches of the split ovarian vein. Missing a diagnosis of OVT in a patient with this anomaly could lead to delayed treatment, subsequently causing thrombophlebitis or sepsis in the patient.

Similarly, in ovarian vein stenosis and pelvic congestion syndrome, imaging modalities such as CT, MRI, and ultrasound, are commonly used to confirm clinical suspicion [4]. A diagnostician should be observant of a fenestrated ovarian vein variant when looking for stenosis or reflux on diagnostic imaging. From a surgical treatment standpoint, these conditions of stenosis and congestion may require the endovascular manipulation of ovarian veins. Stenting or coil embolization of a single side in a fenestrated ovarian vein may not relieve the patient’s ovarian vein stenosis or pelvic congestion, respectively.

## Conclusions

The clinical importance of understanding a variant fenestrated ovarian vein cannot be understated in the medical conditions discussed. Variants have been described previously in the medical literature, although more images of these fenestrated veins would assist clinicians and surgeons in visualizing this anatomical anomaly. The reported image in this case (Figure 1) shows a clear fenestration of the ovarian vein in a cadaver, which can help guide effective treatment plans in female pelvic conditions involving this variation.
